# SELECTIVE ENGAGEMENT IN PREPARATIONS FOR OLD AGE: DETERMINANTS OF MOTIVATION

**DOI:** 10.1093/geroni/igac059.506

**Published:** 2022-12-20

**Authors:** Jeongsoo Park

**Affiliations:** Ajou University, Suwon-si, Kyonggi-do, Republic of Korea

## Abstract

Preparations for old age in general are beneficial for one’s adjustment in later life. Using Selective Engagement Theory (SET) as a conceptual framework, we examined how the importance attached to functioning, as well as perceived control over functioning in different domains (e.g., family, social relations, finances, health, etc.) predicted engagement in preparing for old age five years later. Two-wave data was obtained from Ageing as Future Study. The sample consisted of N = 1,255 aged from 30-85 in the US (n=315), Hong Kong (n=317), and Germany (n=623). Consistent with SET, ratings of importance were strongly predictive of subsequent preparations and more predictive than perceived control, with evidence in several domains of functioning that this was particularly true for older adults. These findings highlight the interaction between personal goals and resources in determining older adults' willingness to prepare for old age.

